# Titanium Dioxide Solar Photocatalytic Microbial Inactivation Assessment Utilizing Viability Tests and a Novel Triplex qPCR Assay for Nucleic Acid Degradation Determination

**DOI:** 10.3390/molecules30224333

**Published:** 2025-11-07

**Authors:** Ioannis Paspaltsis, Eirini Kanata, Sotirios Sotiriadis, Chrysanthi Berberidou, Sophia Tsoumachidou, Athanasios Arsenakis, Konstantinos Xanthopoulos, Dimitra Dafou, Ioannis Poulios, Theodoros Sklaviadis

**Affiliations:** 1 Laboratory of Pharmacology, School of Pharmacy, Faculty of Health Sciences, Aristotle University of Thessaloniki, 54124 Thessaloniki, Greece; ipaspalt@pharm.auth.gr (I.P.); ekanata@pharm.auth.gr (E.K.); sotosotiriadis96@gmail.com (S.S.); xantho@pharm.auth.gr (K.X.); 2 Laboratory of Physical Chemistry, School of Chemistry, Faculty of Sciences, Aristotle University of Thessaloniki, 54124 Thessaloniki, Greece; cberber@chem.auth.gr (C.B.); stsoum@gmail.com (S.T.); poulios@chem.auth.gr (I.P.); 3 Sterimed SA, G’ Fassi, Building Block 52b, Sindos Industrial Area, Thessaloniki 57022, Greece; arsenakis@sterimed.gr; 4 Department of Genetics, Development and Molecular Biology, School of Biology, Faculty of Sciences, Aristotle University of Thessaloniki, 54124 Thessaloniki, Greece; dafoud@bio.auth.gr

**Keywords:** solar, titanium dioxide, photocatalysis, multiplex quantitative PCR, microorganism inactivation efficiency, *Escherichia coli*, *Staphylococcus aureus*, *Candida albicans*, MS2 bacteriophage, *Geobacillus stearothermophilus* spores

## Abstract

Photocatalytic oxidation of microorganisms is a powerful alternative to established disinfection approaches, applicable to a variety of water matrices. Bacterial vegetative cells, spores, fungi, and viruses, represent potential biopathogens and photocatalysis targets. Inactivation efficiency is usually evaluated by assessing viability through culture. However, additional inactivation assessment approaches are needed, as some microbes, despite being unculturable, remain metabolically active and pathogenic. Nucleic acid quantification approaches (qPCR) can assess nucleic acid release and degradation during photocatalysis. We developed a novel multiplex qPCR assay for simultaneous detection/quantification of genomic DNA from different bacterial and fungal species and of MS2 bacteriophage load. Following small-scale solar titanium dioxide photocatalysis on a microbial suspension mixture containing different biopathogen classes, we assessed photocatalytic efficiency by conventional microbiological assays (culture) and our novel molecular assay. Microbiological assays show a significant reduction in microbe viability within one hour of processing, following previously reported patterns of microbial species resistance. Molecular analysis data show that nucleic acids released in solution due to microbial oxidative damage were significantly reduced due to oxidative degradation within six hours. Through targeting different biopathogen classes, our assay could be a useful tool for assessment of photocatalytic microbe inactivation both in laboratory and real-wastewater applications.

## 1. Introduction

Photocatalysis, an advanced oxidation process (AOP), attracts increasing interest in diverse research areas, including the oxidation of hazardous chemicals and the inactivation of microbes. Artificial or solar-emitted photons are utilized by photocatalysts for the generation of reactive oxygen species (ROS) such as O^•−2^ and H_2_O_2_ [1]. The most prominent ROS are the short-lived •OH radicals [2] with a lifetime of 10^−9^ s [3] and a high redox potential of 2.8 eV [4]. Produced ROS oxidize nonspecifically organic molecules (chemical pollutants or microbial components) in proximity to the catalyst. As a result, the toxicity of chemicals or microbial viability/pathogenicity is decreased.

Photocatalysis constitutes a powerful alternative to established detoxification and disinfection technologies in terms of environmental protection and remediation. Different photocatalysts in diverse experimental configurations have shown great potential in reducing toxicity conferred by pesticides, pharmaceuticals, pigments, detergents, organic diluents, and plastics [5,6,7,8,9,10]. Moreover, photocatalytic inactivation of a variety of potential biopathogens has been reported, including different bacterial species, either in the form of vegetative cells or spores, fungi, viruses, or prions [11,12].

TiO_2_ and several TiO_2_-based, performance-enhancing composites [13] are often used for the treatment of chemicals, microbes, or their combination in water [14]. Application of TiO_2_ and TiO_2_ doping for photocatalytic purposes gained popularity because of its important advantages: it is inexpensive, powerful in terms of ROS generation potential, and can be reused without significant loss of action; at the same time, it is environmentally friendly, as it is biologically inert, displaying no known toxicity. Importantly, it can be powered by both artificial and solar irradiation.

Regarding the antimicrobial activity of TiO_2_, substantial research has focused on bacterial inactivation. The initial inactivation step includes adsorption of the bacterial cell on the surface of the catalyst. Irradiation of the catalytic molecule leads to ROS generation, which oxidizes molecules present at proximal bacterial structures. As a result, cell wall or outer cell membrane decomposition is initiated in Gram-positive or Gram-negative bacterial cells, respectively [2,15,16]. Disruption of the outer layer surrounding the bacterium may lead rapidly to bacterial inactivation, resulting in the reduction or complete loss of proliferation ability [17]. A similar microbial particle decomposition pattern, from outside to inside, during photocatalytic treatment, is proposed for other potential biological pathogens, such as fungi and viruses [18,19,20].

Oxidative damage of the cell wall, cell membrane, or viral capsid eventually causes the release of the cellular or viral organic content, which accounts for approximately 96% of microbial dry weight [2], to the surrounding solution. Released biopolymers, such as nucleic acids (DNA and RNA), proteins, lipids, and carbohydrates, as well as organic monomers such as nucleotides, amino acids, and sugar monomers, may be further oxidized as the reaction proceeds. Complete oxidation of organic matter results in photocatalytic mineralization, i.e., the complete decomposition of the organic substances into inorganic compounds [17].

In photocatalytic-treated suspensions, microbial inactivation is usually estimated by classical microbiological methods based on culturing, to assess microbial viability. However, this approach cannot provide precise information related to microbial viability; it was reported four decades ago that microbes under environmental stress, such as the oxidative environment during photocatalytic treatment, may lose their ability to grow on culture media, becoming viable but not culturable [21]. This is a survival strategy, associated with metabolic and genetic changes, resulting in metabolically, transcriptionally, and translationally active, but unculturable cells. Crucially, microbes in this state can retain their pathogenicity, as they express virulence genes; when favorable conditions are restored, they may resuscitate, regaining culturability [22]. Thus, relying exclusively on culture to quantify bacterial density may lead to inaccurate results, posing a significant public health risk, especially when culture methods are used for assessing pathogen inactivation in medical liquid waste before disposal. In this regard, molecular techniques such as quantitative PCR (qPCR), which allow detection/quantification of nucleic acids released from damaged cells and nucleic acid integrity assessment, provide a more detailed and reliable assessment of photocatalysis performance [14].

Thanks to its sensitivity, the qPCR assay may provide accurate data on the degradation state of microbial macromolecules in treated water matrices. This is especially significant when photocatalytic approaches are applied for water remediation or when treated water will be released to the environment. In these cases, any naked DNA derived from dead bacteria may act as a substrate for horizontal gene transfer [23], leading to undesirable gene recombination. Furthermore, through the detection of DNA degradation, the application of qPCR to monitor photocatalytic treatment may provide data on the degradation course of microbes [17]. Since ROS are produced throughout photocatalytic treatment, they oxidize nonspecifically the existing organic matter. However, it is important to note that through qPCR, only DNA fragments larger than approximately 100 bp can be detected; thus, qPCR cannot provide evidence of complete mineralization.

Aiming at providing an accurate molecular tool for assessing photocatalytic efficiency on microbial inactivation, we have developed a novel, hydrolysis probe-based multiplex qPCR assay for the estimation of nucleic acid leakage/damage level during photocatalytic inactivation of microbes. The assay allows the simultaneous detection of bacterial, fungal, and viral genomes. It has been developed for the detection of *Geobacillus stearothermophilus*, *Staphylococcus aureus*, *Escherichia coli*, *Candida albicans*, and the *Emesvirus zinderi* (MS2) bacteriophage. Importantly, the bacterial- and fungal-specific primer–probe sets may detect a wide range of bacterial and fungal species commonly present in urban or medical wastewater, highlighting the potential use of the assay in both laboratory and real-life settings.

Application of the assay on a small-scale solar photocatalytic setting, using TiO_2_/H_2_O_2_ for inactivation of a microbial mixture, provided additional data regarding microbial inactivation. Our data show that microbial viability is markedly reduced within the first hour of photocatalysis, and significant microbial nucleic acid degradation occurs within six hours of treatment. This data indicates that safe estimation of microbial inactivation requires a combination of both culture and molecular assays. The assay developed and evaluated in this study could serve as a molecular tool for the assessment of microbial inactivation under different photocatalysis settings, allowing easier comparison of experimental data obtained under different experimental setups.

## 2. Results and Discussion

Photocatalytic approaches are gaining increasing attention as an efficient, environmentally friendly, and sustainable alternative to traditional disinfection approaches, aiming at the inactivation of various microorganisms, identified as potential biopathogens in water matrices [16,24,25,26,27,28,29,30].

Accurate evaluation of photocatalytic disinfection efficiency is highly needed to ensure safe disposal of treated materials [21,31]. The traditional culture method for the determination of microbial viability is usually applied for assessing photocatalytic efficiency. Molecular assays targeting nucleic acids released from the microorganisms and further degraded by free radicals during photocatalytic treatment, provide an additional and more precise approach for the assessment of photocatalytic efficiency [14,21,32,33,34] and are urgently needed [17].

Aiming to provide a molecular tool for assessing the disinfection efficiency of photocatalytic approaches applied in aqueous matrices, and considering that different water matrices display a wide microbial diversity, we developed and utilized a triplex qPCR assay for the simultaneous detection of representatives from different potential biopathogen classes. Our assay utilizes three primer–probe sets and enables the detection of at least three different bacterial species (set 1), a fungus (set 2), and a bacteriophage (set 3). The assay targets model microorganisms (*Escherichia coli*, *Geobacillus stearothermophilus*, *Staphylococcus aureus*, *Candida albicans*, and MS2), which are widely used for optimization of small-scale photocatalytic approaches in the laboratory [35,36,37] and are also present in real wastewater. Thus, our assay can be utilized in both experimental and real-life settings.

The efficiency of each primer–probe set against the targeted microbes was determined based on standard curves generated by serial dilution of commercial DNA/RNA standards (*Escherichia coli*, *Candida albicans*, MS2). DNA extracted from bacterial cultures of *S. aureus* and *G. stearothermophilus* was also utilized for standard curve preparation. Assay efficiencies were calculated based on curve slopes, using the equation Efficiency = 10^(−1/slope)^ −1. Table 1 summarizes assay efficiencies and detection limits in terms of genome copy numbers per microorganism. Figure S1 displays pertinent standard curves.

**Figure 1:**
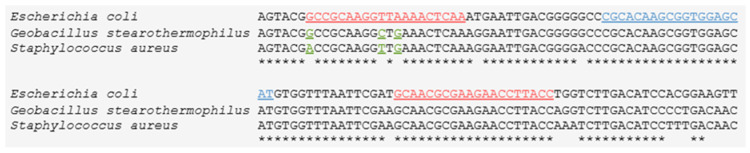
Partial nucleotide alignment sequences from *Geobacillus stearothermophilus* (GenBank AB680491.1), *Staphylococcus aureus* (GenBank NR_118997.2), and *Escherichia coli* (GenBank MW349588.1). The sequences correspond to the 16S rRNA gene and were retrieved from the NCBI nucleotide database. Primer annealing regions are marked in red, the probe region is marked in blue, and mixed bases corresponding to sequence variability among the species are shown in green. The forward primer was designed as a degenerate oligo. Asterisks (*) indicate conserved nucleotide positions shared by all three sequences. Multiple alignment was performed with Clustal omega software (online available at https://www.ebi.ac.uk/jdispatcher/msa/clustalo, accessed on 15 June 2023).

By targeting the highly conserved 16S rRNA and utilizing a degenerate primer (Bact91F, Table 2, Figure 1), our bacteria-specific primer–probe set not only efficiently identifies all three targeted microbes (*Geobacillus stearothermophilus*, *Staphylococcus aureus*, *Escherichia coli*) but also, as indicated by BLASTn analysis, has the potential to identify additional bacteria from other genera, such as *Salmonella*, *Pseudomonas*, *Vibrio*, *Klebsiella*, *Listeria*, *Streptococcus*, and *Enterococcus*, commonly present in water matrices [38,39] (Table S1). Similarly, the fungi-specific primer–probe set targeting 18S rRNA, apart from *Candida*, has the potential to identify additional fungal genera, such as *Fusarium* and *Penicillium*, commonly detected in both urban wastewater [40] and medical liquid waste [41]. These observations highlight the potential versatility of our assay. However, in cases where the targeted microorganisms are other than the ones validated in this study (i.e., *Escherichia coli*, *Candida albicans*, MS2, *S. aureus*, *G. stearothermophilus*), we recommend determining the efficiencies of the corresponding assay primer–probe sets for the microorganisms of interest.

We utilized our multiplex qPCR assay on photocatalytically treated microbial mixture suspensions. We selected TiO_2_ since it is a well-established catalyst, previously used for the photocatalytic inactivation of various microorganisms, including the highly resistant-to-inactivation prion agents [42,43,44,45,46]. Its non-toxicity, high photochemical stability, and potential for reuse further contribute to its wide use as a photocatalyst [47]. TiO_2_ excitation is achieved by irradiation at a wavelength below 375 nm, equivalent to the UV-A range of the spectrum [48], allowing the use of both artificial (UV-A lamps) and natural (solar light) sources of illumination. Considering that electricity and maintenance costs are the major operational costs in artificial UV-A photocatalysis [49], exploitation of solar UV-A light for TiO_2_-mediated photocatalytic disinfection has gained much attention [27,33,42,48,50,51,52]. Targeting a cost-effective and sustainable disinfection approach, we focused on the solar-driven photocatalytic inactivation of microorganisms.

We utilized natural sunlight to treat a mixture of different microbial suspensions (*Geobacillus stearothermophilus* spores, *Staphylococcus aureus*, *Escherichia coli*, MS2, and *Candida albicans*) in the presence of 0.5 g/L TiO_2_ and 500 ppm H_2_O_2_ for 6 h. H_2_O_2_ was added to enhance disinfection efficiency [26,49]. All microorganisms were added at a final concentration of 10^6^ cfu/mL, except for MS2, which was added at 10^8^ pfu/mL. H_2_O_2_ levels were assessed at 60 min, and additional H_2_O_2_ was added to ensure the desired concentration (500 ppm) was maintained throughout the reaction.

Experiments were performed in July, a month of high insolation and high temperature in Greece. Solar UV-A irradiation intensity and temperature were monitored during the experimental process. UV-A irradiation and temperature ranged between 4.1 and 0.31 mW/cm^2^ and 44–30.4 °C, respectively, peaking at the beginning of each experiment and then gradually decreasing.

At predefined time points, samples were collected and used either for microbe viability testing through culture or for nucleic acid isolation for application of our newly developed qPCR assay. Similar reactions, performed under dark conditions, were included in each experiment and served as the negative control.

For the microbial viability tests, samples taken at 0, 15, 30, and 60 min were plated on appropriate agar media using the top agar overlay method. The number of colonies or plaques was determined following 20 h incubation at 37 °C or at 60 °C for *Geobacillus stearothermophilus* spores.

**Figure 2:**
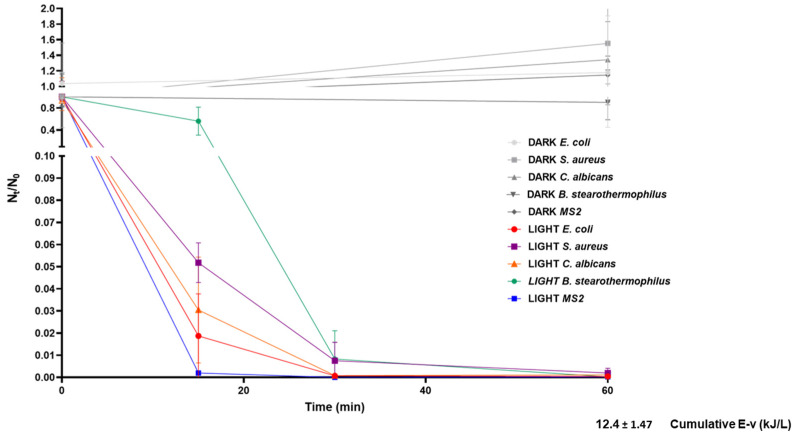
Microbial viability reduction by solar TiO_2_ photocatalysis supplemented with H_2_O_2_ in the tested microbial suspension mixture. The graph depicts viability reduction (N_t_/N_0_) of treated samples and corresponding controls (gray symbols and lines) in relation to processing time and the calculated accumulated energy after 60 min of treatment. The graph summarizes data acquired from two independent experiments (sampling of each time point in triplicate for each experiment). Error bars correspond to standard errors. The legend shows experimental conditions (dark, light) and the tested microorganisms.

We observed a significant reduction in microbial viability within the first hour of treatment. Figure 2 depicts microbial viability data acquired from two independent experiments (sampling of each time point in triplicate for each experiment) in relation to accumulated solar energy (kJ/L). Accumulated energy was determined as previously described.

As shown in Figure 2, we observed microorganism-related differences in terms of inactivation rates; *Geobacillus stearothermophilus* spores displayed the highest resistance to inactivation, followed by *Staphylococcus aureus*, *Candida albicans*, *Escherichia coli*, and MS2 (Figure 2). This pattern is in line with previously described patterns of microbe-specific resistance to inactivation [28], reflecting the impact of individual microorganism characteristics, such as size, structure, and chemical composition. Indeed, spores are the most resistant, due to their compact structure [15], while the MS2 bacteriophage appears to be highly susceptible, possibly due to the lack of genetic material repair mechanisms, which results in higher vulnerability to nucleic acid damage through ROS [29].

Parameters affecting the efficiency of photocatalytic disinfection include catalyst concentration, microorganism load and diversity, composition of treated material, irradiation intensity, and temperature [32]. High catalyst concentrations negatively impact photocatalytic efficiency, resulting in increased turbidity and light scattering [53]. Similarly, microbial load affects the processing time required for disinfection. In addition, disinfection efficiency is affected by the microbe species present in the treated material, and the overall photocatalytic disinfection efficiency of microbial mixtures differs from the inactivation rates of individually tested microorganisms [54]. Moreover, the presence of salts and/or suspended residues, corresponding to components of the treated material, may affect disinfection efficiency [55]. Finally, light intensity and temperature have a considerable impact on disinfection efficiency, with higher light intensities and lower temperatures promoting photocatalysis [49]. Heterogeneity in terms of experimental parameters, including catalyst configuration and concentration, photocatalytic setup, microorganism strains and their initial load, further diversified by fluctuations in light intensity and temperature that characterize outdoor experimentation, hampers a direct comparison of our results with similar published studies.

Nevertheless, we observed the previously reported impact of light intensity and temperature on photocatalytic efficiency. This is depicted in Figure S2, which presents individual data from two experiments conducted under different temperature and solar intensity ranges. Accumulated energy values determined for each experiment are shown. Even though the same pattern of differential microbial resistance to inactivation was observed, the rate of microbial inactivation was altered (Figure S2A,B). Higher inactivation rates were determined under increased accumulated energy and lower temperature (~35 °C), in line with previous reports [49]. Even though not the focus of this study, the effects of photolysis and direct peroxygen chemistry were assessed in a set of experiments through the use of appropriate controls (Supplementary Figure S3).

**Figure 3:**
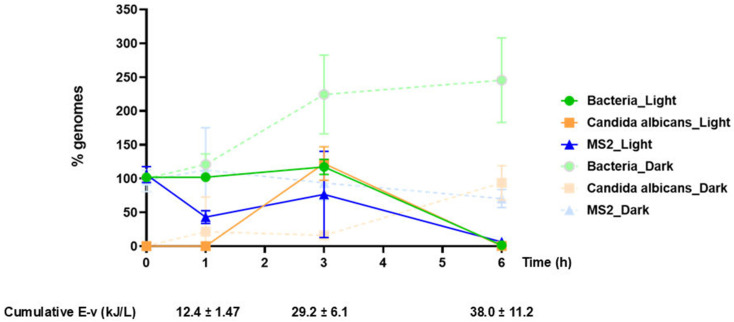
qPCR analysis results of two independent photocatalysis experiments (Light) and their corresponding controls (Dark). The graph shows the percentage of detected genomes at the indicated time points, 0, 1, 3, and 6 h, and in relation to the accumulated energy. For bacteria, values refer to *E. coli* genome equivalents. As a 100% genome was considered the detected microbial genome number at time point 0 h of each condition, except for *C. albicans*, for which the 3 h (Light) and 6 h (Dark) values were assumed to be 100%. Samples of each experiment were analyzed in triplicate.

To gain a better understanding of microbial inactivation during photocatalysis, we next focused on nucleic acid release and degradation, utilizing our newly developed multiplex qPCR method. Tested samples were collected at timepoints 0, 1h, 3h, and 6h, centrifuged to pellet intact bacteria, and the supernatants, harboring DNA released in the solution due to bacterial damage and RNA from both intact and damaged MS2 virions, were processed for DNA/RNA extraction, followed by qPCR. Similar processing was performed for samples under ‘Dark’ conditions. qPCR data were converted to genome equivalents based on corresponding standard curves (Table 1). In the case of bacteria, genome equivalents of *E. coli* were calculated. Table S2 provides detailed qPCR data of two independent experiments for which viability culture assays have been performed (culture assay results shown in Figure 2). Figure 3 depicts results from the corresponding qPCR analyses, expressed as a percentage of nucleic acid signal at each time point relative to an arbitrarily selected reference sample per condition (photocatalytically treated, “Light”, or the dark control, “Dark”) and microbe class (Bacteria, Candida, and MS2).

During photocatalysis, microbial structural elements are oxidized and damaged, leading to nucleic acid release in the solution. This is followed by further oxidation of biological macromolecules by ROS, resulting in nucleic acid degradation. Our molecular assay enables the detection of nucleic acid release and estimation of their integrity through amplification of short nucleic acid fragments (86–110 bp, depending on the assay). In this regard, we expect no detectable bacterial and fungal DNA in the tested supernatant at time point 0, as intact cells are pelleted and not processed. In contrast, the MS2 nucleic acid signal, corresponding to intact virions present in the tested supernatant, is expected to be present at time point 0. As photocatalytic treatment proceeds, compromised microbes and virions release macromolecules, leading to a higher nucleic acid signal. This peak in macromolecule concentration, reflected in the higher nucleic acid level readings, is followed by signal reduction due to nucleic acid fragmentation to the extent that target nucleic acid regions are not intact and the primers can no longer recognize them, initiating their amplification.

Our molecular assay covers a wide range of bacterial species and provides a generalized detection/quantification of bacterial DNA. In our experimentation, this corresponds to DNA from *Geobacillus stearothermophilus*, *Staphylococcus aureus*, and *Escherichia coli*. Our data (Figure 3) showed that in the photocatalytically treated samples, total bacterial DNA decreases significantly after 6 h of treatment (~1.5% of the initial load). A relatively high bacterial DNA signal at time point 0 was observed, which was unexpected, considering that bacterial cells should be intact at this time point. This high initial signal, which is indicative of cellular damage, could be attributed to centrifugation and resuspension of bacterial pellets during the preparation steps. The integrity of *Escherichia coli* and *Staphylococcus aureus* is expected to be affected more severely by this processing than *Geobacillus stearothermophilus* spores, which are compact and thus more resistant to mechanical stress. Bacterial DNA levels, representing DNA released from *Geobacillus stearothermophilus*, *Escherichia coli*, and *Staphylococcus aureus*, remained virtually unaffected for the first 3 h of treatment. The kinetics of the reaction are considered to reflect a dual process, entailing both the oxidation of already released DNA, resulting in signal reduction, and the release of additional DNA from newly damaged bacterial cells during treatment, resulting in signal increases. Considering the results acquired from the corresponding culture assays, we speculate that bacterial DNA release at earlier photocatalysis time points is mostly contributed by the most susceptible bacterial species (*Escherichia coli*), followed by intermediate (*Staphylococcus aureus*) and the most resistant (*Geobacillus stearothermophilus* spores) bacteria. In the corresponding “Dark” samples, the qPCR signal increased with time, probably due to damage related to oxidation caused by the added H_2_O_2_ and mechanical stress related to constant stirring.

*Candida albicans* DNA signal was detected after 3 h of solar illumination, suggesting that a higher treatment time is required for the initial cellular damage of yeast cells, compared to vegetative bacteria (*E.coli*, *S. aureus*). This is in line with previous reports [56] that correlate structural effects of *Candida albicans* to inactivation resistance. Of note, our culture data showed that after 1 h of solar illumination, *Candida albicans* showed practically no growth ability on Sabouraud plates; considering that *Candida albicans* DNA was not detectable until 3 h of treatment, it could be suggested that after 1 h of treatment, *Candida albicans* cells lose their ability to propagate, but remain intact; however, they may retain their metabolic activity and pathogenicity, as previously reported for both *Candida albicans* and other microbes under stress conditions [21,31,57]. Following an additional 3 h of treatment (time point 6 h), *Candida albicans* DNA in the processed supernatants was below the detection limit of our assay (<1 genome equivalent, Table 1), reflecting a highly significant degradation of fungal DNA, indicative of fungal cell damage and thus reduced pathogenicity. Taken together, culture and molecular assay data indicate that a 6 h photocatalytic treatment is preferable to evaluate inactivation of *Candida albicans* and reduced pathogenicity, further highlighting the value of molecular assays for the assessment of microbial photocatalytic inactivation. The “Dark” samples showed, as expected, no *Candida* DNA signal at time point 0, which gradually increased to reach a maximum at 6 h.

RNA from the MS2 virus was isolated directly from the supernatant, corresponding to either RNA from intact or from damaged virions. Considering that the MS2 molecular target assessed by our assay represents one copy/genome, in contrast to the other microbe targets (e.g., *E. coli* target: 7 copies/genome, *G. stearothermophilus* target: 10 copies/genome, *S. aureus* target: 6 copies/genome, fungal target: 21–176 copies/genome, Table 1), we used ×100 more concentrated MS2 (10^8^ pfu/mL) compared to other microbes to ensure efficient signal detection. A significant decrease in MS2 nucleic acid load (down to 10%) was achieved after 6 h of solar irradiation, suggesting significant virion and nucleic acid damage at this time point. On the contrary, no significant signal variations were observed, in the dark control samples.

In summary, we have developed a multiplex qPCR assay allowing the simultaneous detection/quantification of different microbial classes, including bacteria, fungi, and viruses, and utilized it for assessing the inactivation efficiency of a microbe mixture following small, laboratory-scale TiO_2_ solar photocatalysis. Our molecular data provides additional information on the photocatalytic inactivation of different microbial species under solar irradiation, allowing the determination of an efficient processing time in terms of microbe inactivation, based on the estimation of nucleic acid integrity.

Considering that molecular assays contribute to a better estimation of photocatalytic microbial inactivation, our assay, which allows the detection of different microorganism classes, covering a wide range of species commonly detected in urban wastewater and medical liquid waste, could serve as a valuable tool for assessing photocatalytic microbial inactivation in both laboratory and real-life settings. Moreover, widespread assessment of microbial inactivation using the same assay would also allow more efficient comparisons among the results acquired under different experimental settings. In this context, we next plan to use the newly developed multiplex qPCR assay to assess the operation of a novel, much larger (reaction volume 50 L) solar photocatalytic reactor recently developed by our research team.

## 3. Materials and Methods

### 3.1. Multiplex qPCR Assay Development

#### 3.1.1. Primers and Probes

The multiplex assay developed includes three primer-probe sets that target (a) bacteria, (b) fungi, and (c) the MS2 bacteriophage, thus allowing simultaneous detection/quantification of different microbe classes. The bacteria-specific primer–probe set targets the highly conserved region of 16S rRNA, present in all bacterial species used in this study (*Escherichia coli, Staphylococcus aureus*, and *Geobacillus stearothermophilus*). The forward bacteria-specific primer was designed as a degenerate oligo to ensure amplification of all the bacterial species of interest (Figure 1). The fungi-specific primer-probe set targets the 18S rRNA of *Candida albicans* (designed based on the reference sequence GenBank AF114470.1), while the MS2 primer–probe set was designed (based on the reference sequence GenBank NC_001417.2) to target an 86 bp region within the gene encoding for the viral capsid protein.

All oligonucleotides were custom-designed by the Primer3web online tool, version 4.1.0, https://primer3.ut.ee/ (accessed on 13 June 2023) [58] and synthesized by Integrated DNA Technologies (IDT). Primers and probes were analyzed with the BLASTn tool [https://blast.ncbi.nlm.nih.gov/Blast.cgi, [59]] (accessed on 15 July 2023) to ensure specificity to the target genome. Possible homo- and heterodimer formations, GC content, and secondary structure were evaluated using the online OligoAnalyzer tool (Integrated DNA Technologies, IDT), https://www.idtdna.com/calc/analyzer (accessed on 8 June 2023). All probes were HPLC-purified and labeled at the 5′ end with a specific fluorophore: 6-FAM for bacterial targets (with double quenching using ZEN as an internal quencher), ATTO550N for yeast, and Cy-5 for MS2. Primer and probe sequences are listed in Table 1.

A BLASTn analysis of the bacterial primer–probe sets showed a 100% sequence identity with various bacterial species due to the conservation of the targeted region. Similarly, several fungal species are recognized by the fungi-targeting primer-probe set. A summary of selected bacterial and fungal species targeted by the designed primer-probe sets is shown in Table S1.

#### 3.1.2. qPCR

Reactions were set in a final volume of 20 μL, containing appropriate DNA/cDNA amounts (serial dilutions of standards for standard curve preparation, isolated nucleic acids at indicated time points after photocatalytic treatment, or the corresponding dark control samples). Primer concentration for *C. albicans* and MS2 genome detection was 300 nM, while the concentration of the bacteria-specific Bac91F and Bac91R primers was 450 nM. Each hydrolysis probe was used at a concentration of 200 nM. Each reaction contained 10 μL of master mix (final concentration in reaction 1×), supplemented with the reference dye ROX (2× Luna Universal Probe qPCR Master Mix, New England Biolabs, M3004). Thermal cycling was performed in an Applied Biosystems 7500Fast real-time PCR system (Applied Biosystems, Waltham, MA, USA). Cycling conditions included an initial denaturation step at 95 °C for 2 min followed by 40 cycles of 95 °C for 15 s, 60 °C for 30 s, and 68 °C for 20 s.

All samples were tested in triplicate, and appropriate negative controls (no template reactions) were included in each run.

#### 3.1.3. DNA/RNA Standards, Standard Curves and Reaction Efficiency Estimation

The following commercial nucleic acid standards were purchased from Merck & Co., Inc. (Rahway, NJ, USA):: (a) MS2 phage RNA standard (10165948001, 800 ng/μL), (b) fungal DNA standard from *Candida albicans* (MBD0044-0.3UG, 10 ng/μL), (c) microbial DNA standard from *E. coli* (MBD0013-0.3UG, 10 ng/μL), and used for standard curve preparation for assay efficiency estimation and subsequent microbe quantification. The MS2 RNA standard was reverse transcribed to cDNA using the Τakara PrimeScript RT Reagent Kit (TAKARA Bio Inc, Kusatsu, Shiga, Japan) (RR037A) according to the manufacturer’s instructions.

To assess the primer-probe efficiencies, standards were serially diluted (1:20 and then four additional 1:10 dilutions) and used for the preparation of standard curves. Reaction efficiencies were determined from the acquired curves using the equation Efficiency = 10^(−1/slope)^ − 1. For the determination of the limit of detection, the following equation was used: LOD = (3.3 × standard deviation of linear regression)/slope of the regression line (standard deviation of linear regression = Sy.x).

### 3.2. Solar Titanium Dioxide Photocatalytic Inactivation of Microbial Mixture Suspensions

#### 3.2.1. Microbial Strains

The microbial strains used in this study were selected to include representatives of all potential biopathogen classes, namely bacterial spores, fungi, viruses, and Gram-positive and Gram-negative bacteria. Model organisms, including *Geobacillus stearothermophilus* (ATCC 7953) endospores, *Staphylococcus aureus* (ATCC 6538), and MS2 bacteriophage (ATCC 15597-B1), were used. In addition, a clinical isolate of the yeast *Candida albicans* and an ampicillin-resistant laboratory strain of *Escherichia coli* (TOP10 transformed with a plasmid vector carrying ampicillin resistance) were utilized.

*Bacillus* spores were used as a representative of species highly resistant to inactivation. *Staphylococcus aureus* and *Escherichia coli* were used as representatives of Gram-positive and Gram-negative bacteria, respectively, and *Candida albicans* as a fungal representative. The MS2 bacteriophage, displaying morphological and genomic similarities to human enteric viruses (RNA genome, size, and shape), was used as a non-pathogenic viral surrogate.

Microbial strains were kindly provided by Emeritus Professor Minas Arsenakis (School of Biology, Aristotle University of Thessaloniki, Thessaloniki, Greece).

#### 3.2.2. Microbial Propagation and Culture Assays

Microorganisms were propagated in appropriate or selective nutrient media (broth or agar). Tryptic Soy Broth Agar-TSB (Applichem, 413820) was used at 60 °C for *Geobacillus stearothermophilus*, Sabouraud Dextrose Agar—SDA (dextrose 40 g/L, peptone 10 g/L, agar 15 g/L, pH 5.6) was used at 37 °C for *Candida albicans*; Luria–Bertani-LB (10 g/L tryptone, 5 g/L yeast extract, and 5 g/L NaCl) supplemented with 100 μg/mL ampicillin was used at 37 °C for *Escherichia coli* (TOP10 transformed with an ampicillin resistance conferring plasmid); and Mannitol Salt Agar—MSA (peptone 10 gr/L, sodium chloride 75 gr/L, D-mannitol 10 g/L, meat extract 1g/L, agar 15 g/L)—was used at 37 °C as a selective for *Staphylococcus* species medium due to its high salt concentration. The MS2 host strain (*E. coli* Top10F’) was propagated in Luria–Bertani-LB.

Culture assays for microbe viability estimation were performed on appropriate agar plates by overlaying corresponding top agar media (7 g/L of agar) to ensure homogeneous distribution of microbes. *Geobacillus stearothermophilus* endospores were processed as previously described [42].

Plates were incubated at 37 °C (*S. aureus, E. coli*, *C. albicans*, MS2) or 60 °C (*G. stearothermophilus*) for 20 h, and colonies/plaques were counted to determine colony/plaque-forming units.

#### 3.2.3. Solar Photocatalytic Treatment of Microbial Mixture Suspensions

Photocatalysis was conducted using titanium dioxide (TiO_2_) P25 (Aeroxide, Evonik, (Essen, Germany); 70% anatase–30% rutile) and solar light as the irradiation source. Treatment was performed in Phosphate-Buffered Saline (PBS) under continuous stirring (400 rpm), in 6-well plates (Greiner, 657185), at a final volume of 10 mL per well, containing 0.5 g/L TiO_2_ and 500 ppm H_2_O_2_ per well. Freshly prepared cultures of *E. coli* and *S. aureus* at the exponential phase (OD600 = 0.5) and overnight *C. albicans* cultures (OD600~ 1.3) were used. Cultures at the desired growth phase were left on ice for 10 min to stop microbial growth, centrifuged (2500× *g*/20 min/4 °C), and resuspended in PBS. Previously prepared and titrated stocks of MS2 (10^11^ pfu/mL) and *G. stearothermophilus* spores (2 × 10^7^ cfu/mL) were used. All microorganisms were added at a final concentration of 10^6^ cfu/mL except MS2, which was added at a higher concentration (10^8^ pfu/mL). We used ×100 more concentrated MS2 (10^8^ pfu/mL) compared to other microbes to ensure efficient signal detection, taking into account that the MS2 molecular target assessed by our assay represents one copy/genome, in contrast to the other microbe targets (e.g., bacterial target: 7 copies/genome, fungal target: 21–176 copies/genome, Table 1) and considering the genome copy numbers detection limits per microbial class (Table 1).

At different time points (0, 15, 30, 60, 180, and 360 min), samples were collected and used either for determining residual viability by plating, utilizing the top agar overlay method (timepoints 0, 15, 30, and 60 min), or for extracting nucleic acids for use as templates in subsequent multiplex qPCR assays (timepoints 0, 60, 180, and 360 min). H_2_O_2_ was added at a final concentration of 500 ppm immediately after sampling at t = 0.

Collected samples were plated on appropriate nutrient media, incubated overnight, and the colonies/plaques were counted to determine residual viability. For *G. stearothermophilus* endospores, samples were boiled for 10 min to stimulate germination, mixed with 3 mL of TSB soft agar, overlayed onto TSB agar plates, and incubated overnight at 60 °C. All samples were plated in triplicate.

Solar UV-A intensity (mW/cm^2^) was measured with a photometer/radiometer (UV-1700, PharmaSpec, Shimadzu, Japan), and temperature was monitored during the experiments. H_2_O_2_ concentration was evaluated at t = 60 min using indicator strips (MQuant, Merck,1.10337.0001) and supplemented to 500 ppm. Accumulated solar energy was calculated as previously described [60].

In parallel, control reactions were conducted, containing the catalyst, H_2_O_2_, and the microbial mix suspension, in the dark. Even though not the focus of this study, the effects of photolysis and direct peroxygen chemistry were assessed in a set of experiments through the use of appropriate controls (Supplementary Figure S3).

#### 3.2.4. Microbial Viability Estimation Through Culture Assays

Microbial culture assays were used to quantify the inactivation of microbes during the photocatalytic treatment. Viability reduction over photocatalytic time was quantified using the ratio N_t_/N_0_, where N_t_ represents the number of viable and culturable cells/virus-like particles (cfu/mL or pfu/mL) at specific time points, and N_0_ corresponds to initial content at time point zero. The inactivation efficiency was estimated for samples (s) and time points based on the colony or plaque counts relative to the zero time point (N_ts_/N_0s_).

#### 3.2.5. Nucleic Acid Purification from Photocatalytically Treated Samples

Samples were collected at defined time points (0, 1, 3, 6 h), centrifuged for 15 min, 3000× *g* at 4 °C to remove TiO_2_, cells, and cellular debris, and the supernatant, containing free DNA/RNA and MS2 viral particles, was transferred to new tubes and stored at −20 °C until further processing for nucleic acid purification.

A total of 400 μL of the supernatant was used for DNA recovery using linear polyacrylamide (LPA) as a carrier [61] (France). Briefly, 8 μL of 0.25% LPA was added to each sample. Following the addition of 2.5 volumes of absolute ethanol, samples were incubated for 15 min at –80 °C, centrifuged for 15 min at 20.000× *g* at 4 °C, washed with 70 % ethanol, and centrifuged again under the same conditions. Pellets were air-dried and resuspended in 30 μL of water for injection.

For RNA extraction, 400 μL of supernatant was used. MS2 RNA was extracted using the NucleoSpin RNA virus kit (Macherey-Nagel, Düren, Germany, 740956.50) according to the manufacturer’s instructions. Isolated RNA was finally eluted in 30 μL of elution buffer. Viral RNA was reverse transcribed using the LunaScript RT SuperMix (New England Biolabs, Ipswich, MA, USA, E3010). First strand cDNA synthesis reaction contained 8 μL of RNA and 2 μL of the LunaScript RT SuperMix

1 μL of the isolated DNA and 1.25 μL of the prepared cDNA were applied as template in each single PCR reaction as described in Section 3.1.2.

## Figures and Tables

**Table 1:** Amplification efficiencies and detection limits determined for each of the targeted microorganisms by the newly developed multiplex qPCR assay.

Target Microorganism	Genome Size (kbp)/Target Gene Copies in Genome	Standard Curve Equation/R2	Assay Efficiency **	Standard Deviation of Linear Regression	Genome Copy Number Detection Limit ^¶^
MS2 ^#^	3569/ 1	Y = −3.303X + 40.55 (R2 = 0.9974)	100.8	0.3551	1 (0.4)
*Candida albicans* ^#^	16,000/ 21–176 ^¥^	Y = −3.821X + 33.69 (R2 = 0.9972)	82.7	0.2951	1 (0.56)
*Escherichia coli* ^#^	~5000/ 7 **	Y = −3.308X + 33.86 (R2 = 0.996)	100.6	0.4394	1 (0.08)
*Staphylococcus aureus* (ATCC 6538) ^$^	2,800,485/ 5	Y = −3.539X + 38.30 (R2 = 0.9994)	91.675	0.1811	2 (1.47)
*Geobacillus stearothermophilus* (ATCC 7953) ^$^	2,787,229/ 10	Y = −3.476X + 40.04 (R2 = 0.9988)	93.949	0.2432	2 (1.70)

^#^ Assay efficiency determined by construction of standard curves using commercially available nucleic acid standards. ^$^ Assay efficiency determined by construction of standard curves using DNA corresponding to predetermined genome copy numbers. ** Assay efficiency was determined based on the slope of the corresponding standard curve using the equation Efficiency = 10^(−1/slope)^ − 1; ^¶^ Genome copy number detection limit was determined using the equation LOD = (3.3 × standard deviation of linear regression)/slope of the regression line, standard deviation of linear regression = Sy.x; ^¥^ Michael L Pendrak (DOI: 10.1261/rna.028050.111); ECOCYS database, https://www.biocyc.org/gene?orgid=ECOLI&id=RRSA-RRNA, accessed on 25 August 2025.

**Table 2:** Oligonucleotide sequences of primers and probes. FAM, 6-carboxyfluorescein; IBFQ, 3′ Iowa Black Fluorescent Quencher; IBRQ, 3′ Iowa Black Red Quencher; Cy5, Cyanine5. R = G or A, Y = C or T.

Target	Target Gene	Oligo Name	Sequence (5′—>3′)	Binding Site	Length (bp)
Bacterial gDNA	16S rRNA	Bact91F	RCCGCAAGGYTRAAACTCAA	880–899 ^1^ 901–920 ^2^ 806–825 ^3^	20
		Bact91R	GGTAAGGTTCTTCGCGTTGC	951–970 ^1^ 974–993 ^2^ 877–896 ^3^	20
		Bact91probe	5-FAM/CGC ACA AGC/ZEN/GGT GGA GCA T/IBFQ	917–935 ^1^ 940–938 ^2^ 843–861 ^3^	19
Fungal gDNA	18S rRNA	18S C.al 110F	TAGTTGAACCTTGGGCTTGG	522–541	20
		18S C.al 110R	CAAAGTAAAAGTCCTGGTTCGC	610–631	22
		18S C.al 110probe	ATTO550N/CTGGACCCAGCCGAGCCTTT/IBRQ	569–588	20
MS2 cDNA	Capsid protein	MS2 86F	AATCAGGCAACGGCTCTCTA	1287–1306	20
		MS2 86R	TTGTCGACGAGAACGAACTG	1353–1372	20
		MS2 86pr	Cy5/AGA GCC CTC AAC CGG AGT TTG AAG/IBRQ	1309–1333	24

Binding sites of oligos at reference sequences mentioned above: ^1^ *Geobacillus stearothermophilus*, ^2^ *Staphylococcus aureus*, ^3^ *Escherichia coli*.

## Data Availability

The original contributions presented in this study are included in the article/Supplementary Material. Further inquiries can be directed to the corresponding author(s).

## Supplementary Materials

The following supporting information can be downloaded at: https://www.mdpi.com/article/10.3390/molecules30224333/s1, Figure S1: Assay performance against the targeted microbes. Graphs depict standard curves constructed using serial dilutions of commercially available nucleic acid standards. DNA extracted from bacterial cultures, corresponding to predetermined genome copy numbers, was also used for standard curve construction in the case of *S. aureus* and *G. stearothermophilus*. Assay efficiency in each case was determined from the slope of the corresponding standard curve. Figure S2: The effect of solar UV-A intensity and temperature on photocatalytic inactivation of the tested microbial mixture. The graphs depict viability reduction (N_t_/N_0_) of treated samples and corresponding controls (gray symbols and lines) in relation to accumulated energy and processing time for two independent experiments (A, B, sampling of each time point in triplicate for each experiment). Error bars correspond to standard errors. The legends show experimental conditions (dark, light) and tested microorganisms. Higher accumulated energy was associated with higher inactivation rates (A). Figure S3: Culture and molecular data corresponding to the tested (A) bacteria (*E.coli*, *S. aureus*, *G. stearothermophilus*), (B) fungi (*C. albicans*), and (C) virus (*MS2* bacteriophage). TiO_2_ solar light driven photacatalysis in the presence of H_2_O_2_ was applied to a mixture of all tested microorganisms; controls corresponding to solar light-only (microorganisms under the solar light without addition of TiO_2_ and H_2_O_2_), TiO_2_-only (microorganisms in the presence of TiO_2_, without H_2_O_2_, in dark) and H_2_O_2_-only (microorganisms in the presence of H_2_O_2_, without TiO_2_, in dark) were included to evaluate the contribution of photolysis (solar light), TiO_2_ effects (TiO_2_-only) and peroxygen chemistry (H_2_O_2_-only). Samples were collected at defined time points (0, 60, 180, 270 min) and used for plating on appropriate culture media for evaluation of viable microorganisms (cfu/mL, pfu/mL, microbiological assay, left side panels) and for DNA/RNA extraction applied on molecular analysis utilizing our newly developed qPCR assay (right side panels). The graphs on the left present the % ratio of culturable microorganisms at each time point relative to corresponding initial counts (% N_t_/N_0_). The graphs on the right present relative genome copy number changes during the experiment, expressed as % genome copy number change relative to the highest signal acquired in each case. The *x*-axis presents accumulative Energy (kJ/L), which was determined considering (i) UV-A measurements during experimentation, (ii) treated volume, and (iii) irradiated surface [61]. Sampling time points (min) are also depicted below the cumulative energy values. The relation to the Energy *X*-axis is not applicable to the TiO_2_ and H_2_O_2_ controls, as they were performed in the dark. TiO_2_ and H_2_O_2_ controls are shown in dashed lines to highlight that for these, only time associations are applicable. Since our molecular assay does not discriminate among the tested bacterial species (A, right panel), culture data in panel A left correspond to cumulative data. Data presented correspond to triplicate, and error bars represent standard errors. Table S1: List of selected microbial species with 100% sequence identity to the designed primers and probes, based on BLASTn analyses. Table S2: qPCR data from two independent photocatalytic experiments. The table shows data from photocatalytically treated (“Light”) microbes at four time points (0, 1, 3 and 6h) and corresponding controls (“Dark”) which were not illuminated. Genome equivalents were calculated according to the equations derived from the standard curves using commercially obtained nucleic acid standards. In the case of bacteria, data refers to genome equivalents of *E. coli*. StDev: standard deviation.

## Abbreviations

The following abbreviations are used in this manuscript:

qPCR	Quantitative Polymerase Chain Reaction
cfu	Colony-Forming Units
pfu	Plaque-Forming Units
AOP	Advanced Oxidation Process
ROS	Reactive Oxygen Species
TiO_2_	Titanium Dioxide
HPLC	High-Performance Liquid Chromatography
TSB	Tryptic Soy Broth
SDA	Sabouraud Dextrose Agar
LB	Luria–Bertani
MSA	Mannitol Salt Agar
PBS	Phosphate-Buffered Saline
LPA	Linear Polyacrylamide
