# Supplementary material for: Titanium Dioxide Solar Photocatalytic Microbial Inactivation Assessment Utilizing Viability Tests and a Novel Triplex qPCR Assay for Nucleic Acid Degradation Determination
Source: Molecules. 2025 Nov 7;30(22):4333. doi: 10.3390/molecules30224333 (PMC12654508; doi:10.3390/molecules30224333)
Supplement: Supplementary file 1 [file molecules-30-04333-s001.zip › molecules-3879061-supplementary/Table S1.pdf]

Table S1. List of selected microbial species with confirmed 100 % sequence identity to the designed primers and probes, based on BLASTn analysis performed against NCBI reference genomes.

Taxus	Genus	Species	Acc. No (GenBank)
**Bacteria**	*Escherichia*	*Escherichia coli*	CP149449.1
	Geobacillus	*Geobacillus stearothermophilus*	AB680491.1
	Staphylococcus	*Staphylococcus aureus*	GCF_000705495.1
		Staphylococcus epidermidis	AP028322.1
		Staphylococcus capitis	CP134823.1
		Staphylococcus haemolyticus	CP142094.1
	Salmonella	*Salmonella enterica*	CP150140.1
	Pseudomonas	*Pseudomonas aeruginosa*	CP145538.1
		Pseudomonas canadensis	CP145491.1
		Pseudomonas aeruginosa	CP143907.1
	Vibrio	*Vibrio parahaemolyticus*	CP149505.1
	Klebsiella	*Klebsiella pneumoniae*	CP149487.1
	Listeria	*Listeria ivanovii*	CP149497.1
	Streptococcus	*Streptococcus pyogenes*	CP147828.1
		Streptococcus thermophilus	CP142105.1
	Enterococcus	*Enterococcus faecium*	AP027233.1
	Lactobacillus	*Lactobacillus delbrueckii*	CP142106.1
	Aeromonas	*Aeromonas jandaei*	CP149571.1
	Moraxella	*Moraxella canis*	CP139961.1
	Coprococcus	*Coprococcus comes*	CP143955.1
**Fungi**	*Candida*	*Candida albicans*	CP025165.1
		Candida orthopsilosis	CP084605.1
		Candida parapsilosis	OZ022550.1
	Debaryomyces	*Debaryomyces hansenii*	CP046879.1
	Fusarium	*Fusarium graminearum*	CP079831.1
		Fusarium pseudograminearum	CP103000.1
		Fusarium proliferatum	CP128301.1
	Penicillium	*Penicillium digitatum*	CP060774.1
	Saccharomycopsis	*Saccharomycopsis fibuligera*	CP095750.1
	Trichoderma	*Trichoderma atroviride*	CP084939.1
		Trichoderma asperellum	CP084943.1
	Zymoseptoria	*Zymoseptoria tritici*	CP051576.1
		Zymoseptoria tritici	CP051556.1
	Epichloe typhina	*Epichloe typhina*	CP064799.1
