# Supplementary material for: Titanium Dioxide Solar Photocatalytic Microbial Inactivation Assessment Utilizing Viability Tests and a Novel Triplex qPCR Assay for Nucleic Acid Degradation Determination
Source: Molecules. 2025 Nov 7;30(22):4333. doi: 10.3390/molecules30224333 (PMC12654508; doi:10.3390/molecules30224333)
Supplement: Supplementary file 1 [file molecules-30-04333-s001.zip › molecules-3879061-supplementary/Table S2.pdf]

Table S2: qPCR data from two independent photocatalytic experiments each one analyzed in triplicates. Table shows data from photocatalyticaly treated ("Light") microbes at four time points (0, 1, 3 and 6h) and corresponding controls ("Dark") which were not illuminated. Genome equivalents were calculated according to the equations derived from the standard curves using commercially obtained nucleic acid standards. In the case of bacteria, data refer to genome equivalents of E. coli. StDev: standard deviation.

**Microbial class**	**Time point / Condition**	**Genome equivalents**	**Percentage (%)**	**% average/ StDev**
**Bacteria**	0h / Light	173945<br>178854<br>165675<br>29909<br>32284<br>31397	100<br>102.8<br>95.2<br>100<br>107.9<br>104.9	101.8/<br>4.4
	1 h / Light	161127<br>183903<br>172738<br>30535<br>31397<br>32062	92.6<br>105.<br>99.<br>102.1<br>104.9<br>107.1	101.7/<br>5.3
	3 h / Light	194.431<br>217.330<br>221.915<br>29908<br>33197<br>38150	111.7<br>124.9<br>127.5<br>99.9<br>110.9<br>127.5	117.1/<br>11.3
	6 h / Light	2.711<br>2.787<br>2.600<br>434	1.5<br>1.6<br>1.4<br>1.4	1.4/<br>0.1

		428<br>388	1.4<br>1.3	
	0 h /<br>Dark	94947<br>94947<br>91701<br>30967.<br>30116<br>32968	100<br>100<br>96.5<br>100<br>97.2<br>106.4	100/<br>3.5
	1 h /<br>Dark	109125<br>119458<br>118500<br>37368<br>44463<br>29696	114.9<br>125.8<br>124.8<br>120.6<br>143.5<br>95.8	120.9/<br>15.6
	3 h /<br>Dark	256834<br>256834<br>275343<br>54025<br>52917<br>52553	270.5<br>270.5<br>289.9<br>174.4<br>170.8<br>169.7	224.3/<br>58.14
	6 h /<br>Dark	249783<br>279202<br>281151<br>62965<br>86119<br>42943	263.1<br>294.6<br>296.1<br>203.3<br>278.1<br>138.6	245.6/<br>62.5
Candida albicans	0h /<br>Light	0<br>0	0<br>0	0/<br>0

		0<br>0<br>0<br>0	0<br>0<br>0<br>0	
	1 h /<br>Light	0<br>0<br>0<br>0<br>0<br>0	0<br>0<br>0<br>0<br>0<br>0	0/<br>0
	3 h /<br>Light	1534<br>2230<br>2454<br>1298<br>1510<br>1451	100<br>145.3<br>159.9<br>100<br>116.3<br>111.7	122.2/<br>24.8
	6 h /<br>Light	0<br>0<br>0<br>1<br>1<br>0	0<br>0<br>0<br>0<br>0<br>0	0/<br>0
	0 h /<br>Dark	0<br>0<br>0<br>0<br>0<br>0	0<br>0<br>0<br>0<br>0<br>0	0/<br>0
	1 h /<br>Dark	145<br>166	0.5<br>0.1	0.5/<br>0.4

		183<br>966<br>1319<br>624	0.1<br>0.8<br>1.2<br>0.5	
	3 h / Dark	3863<br>4423<br>4020<br>17674<br>18538<br>17120	14.9<br>17.1<br>15.5<br>16.1<br>16.9<br>15.6	16.0/ 0.8
	6 h / Dark	25899<br>38869<br>21057<br>109412<br>149246<br>89667	100<br>150.1<br>81.3<br>100<br>136.4<br>81.9	108.2/ 28.6
**MS2**	0h / Light	3043640<br>3379154<br>3426599<br>212226<br>259776<br>188495	100<br>111<br>112.5<br>100<br>122.4<br>88.8	105.8/ 110.8
	1 h / Light	1433534<br>1730431<br>1443562<br>87558<br>72527<br>66252	47.1<br>56.8<br>47.4<br>41.2<br>34.1<br>31.2	42.9/ 9.4
	3 h / Light	4253243	139.7	76.6/

		4165213<br>3857742<br>67173<br>18758<br>34158	136.8<br>126.7<br>31.6<br>8.8<br>16.1	63.8
	6 h /<br>Light	375924<br>368144<br>338599<br>1263<br>1840<br>1178	12.3<br>12.1<br>11.1<br>0.5<br>0.8<br>0.5	6.2/<br>6.1
	0 h /<br>Dark	2959939<br>2610870<br>2818962<br>184586<br>227562<br>135831	100<br>88.2<br>95.2<br>100<br>123.2<br>73.5	96.6/<br>16.3
	1 h /<br>Dark	1659542<br>1671152<br>1636564<br>329230<br>302830<br>307043	56.1<br>56.4<br>55.2<br>178.3<br>164<br>166.3	112.7/<br>62.4
	3 h /<br>Dark	3107966<br>3064933<br>3218211<br>170962<br>78867	105<br>103.5<br>108.7<br>92.6<br>42.7	93.4/<br>25.5

		199296	107.9	
	6 h /<br>Dark	2592731<br>2031375<br>2132964<br>152932<br>105681<br>100646	87.5<br>68.6<br>72<br>82.8<br>57.2<br>54.5	70.4/<br>13.2
