# Supplementary figures and images for: Titanium Dioxide Solar Photocatalytic Microbial Inactivation Assessment Utilizing Viability Tests and a Novel Triplex qPCR Assay for Nucleic Acid Degradation Determination
Source: Molecules. 2025 Nov 7;30(22):4333. doi: 10.3390/molecules30224333 (PMC12654508; doi:10.3390/molecules30224333)

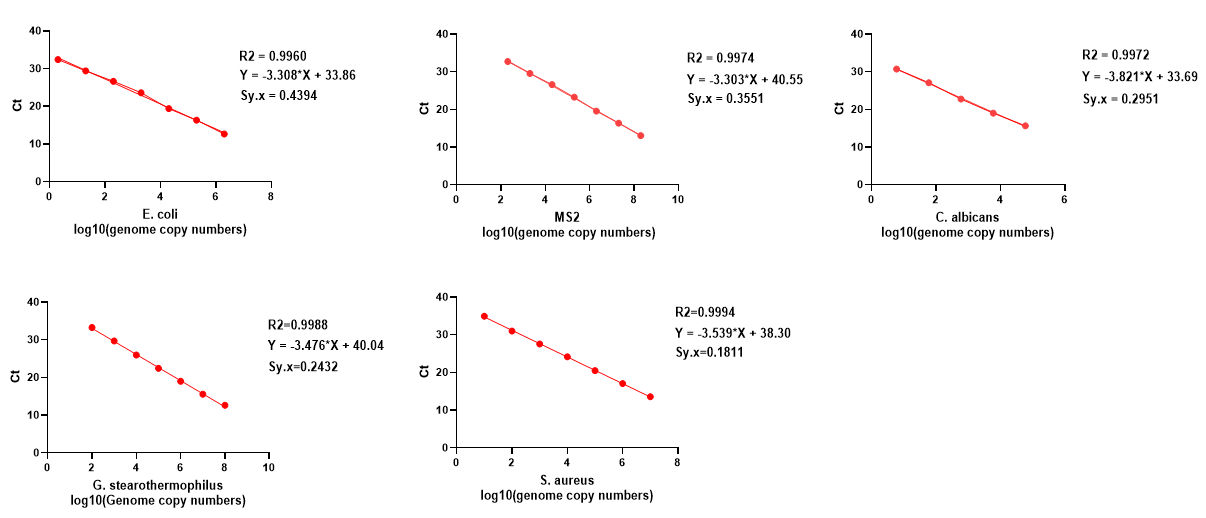

Supplement: Supplementary file 1 [file molecules-30-04333-s001.zip › molecules-3879061-supplementary/Figure S 1.png]

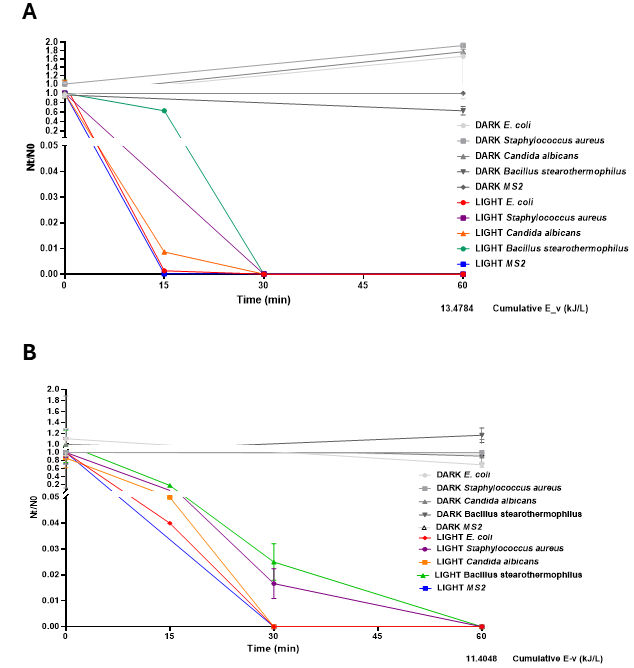

Supplement: Supplementary file 1 [file molecules-30-04333-s001.zip › molecules-3879061-supplementary/Figure S 2.png]

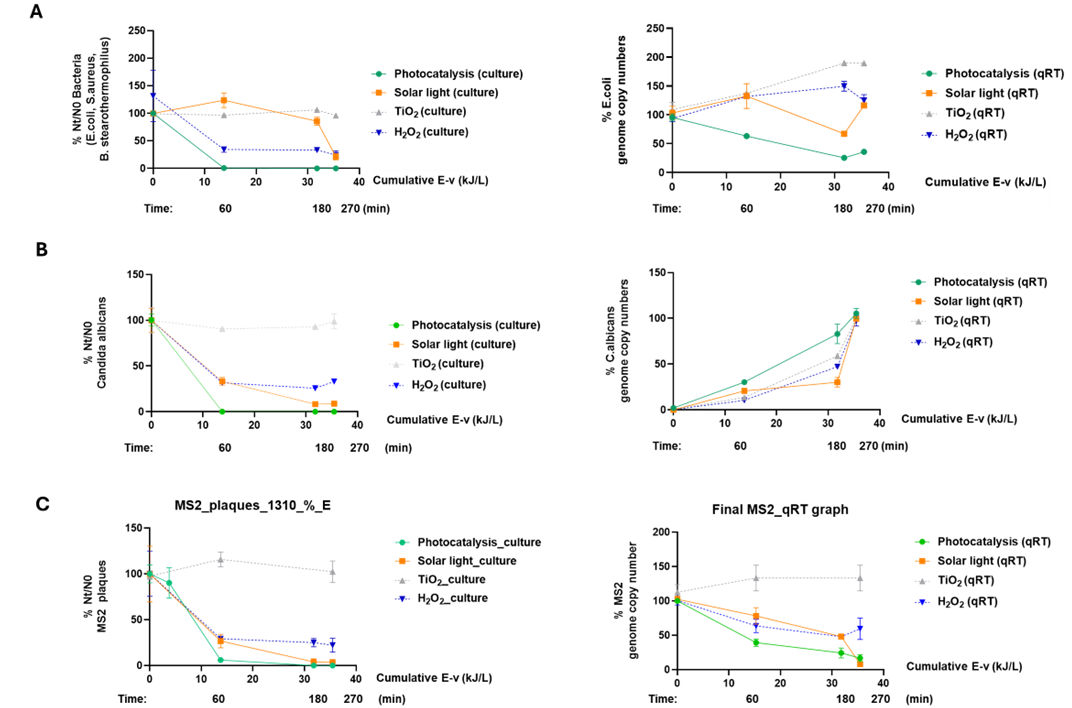

Supplement: Supplementary file 1 [file molecules-30-04333-s001.zip › molecules-3879061-supplementary/Figure S 3.png]
